# Notes on Celluloid

**Published:** 1875-06

**Authors:** James B. Hodgkin

**Affiliations:** Prof. of Dental Mechanism and Metallurgy in the Baltimore College of Dental Surgery.


AMERICAN JOURNAL

OF

DENTAL SCIENCE.

Vol. IX. THIRD SERIES—JUNE, 1875. No. 2.

### ARTICLE I.

### *Notes on Celluloid.*—Continued.

BY JAMES B. HODGKIN, D. D. S.

Prof. of Dental Mechanism and Metallurgy in the Baltimore College of Dental Surgery.

As was stated in the last number of this Journal, the best method of working Celluloid has not yet been discovered; it seems probable that the "dry method" has decided advantages over any other in one important respect—that of developing the welding properties of this substance. Oil or glycerine will entirely prevent even a partial union of the surfaces brought in contact, and in steam the chances would not seem so great of a good joint, though no experiments have been made so far in this direction. With the dry process however, the advantages are manifest, as there is no fluid interposed to prevent union.

Notwithstanding these advantages in favor of welding Celluloid, it is doubtful if so far the results indicate that a perfectly strong and solid joint can be made, one in which perfect union of the parts takes place. It is thought by some that the dry process favors welding to some extent, because the free camphor present in the plate acts as a solvent of the heated Celluloid, and so facilitates the flowing together. But several experiments seem to indicate that this adherence is not to be depended on. Several pieces of Celluloid were pressed into a mould, so constructed that each piece in its passage was cut through by a thin and narrow saw blade, the cut surfaces passing below the blade and coming together were strongly forced into contact while in a heated and plastic condition. On being removed from the flask, none of the pieces were sufficiently strongly united to stand the strain of bending, the strips snapping assunder on quite a moderate strain being applied, while one was not united at all. This experiment seemed to settle the question of the union of surfaces that have been cut by passing over pins or loops, and it seems probable that when Celluloid passes over a pin in a tooth that it does not firmly unite behind it, and only holds the tooth by its being moulded closely around it, which indeed is sufficient to hold most teeth in position.

Other experiments with a view of testing the welding properties of Celluloid with the aid of camphor as a solvent of the surfaces to be united, were made with only moderately satisfactory results. In one of these cases the plate to which it was desired to attach a new piece,—the case was one in which a tooth had been cut off a plate with a view of resetting it in a slightly altered position, was inverted, as for repairing with rubber, and the part to be operated on was scraped clean and painted with a solution of camphor in alcohol. This instantly converted the surface into a semi-pasty substance, seemingly highly favorable to union with the piece to be welded. The patch to be united was painted in the same manner with the camphor solution, placed in position, the flask heated and closed. This piece seemed perfectly united and the result looked highly favorable, but on finishing it up, the joint was found to be not at all strong, breaking on the application of a force not sufficient to slightly spring the thin plate. This experiment was repeated several times, in various ways with the same result, establishing the conviction that two surfaces of Celluloid do not weld perfectly under the methods of manipulation at present known. Further research in this direction is earnestly to be desired. When it is remembered how long we were without the simple and magical cement known as Rubber Solder, and how "dove tails" and holes were cut to mechanically retain the new rubber in position, the success which Celluloid has achieved is remarkable.

Such observations as we have been able to make of the action of camphor leads to one or two conclusions, the first of which is that a surface wet with camphor solution is expanded by it, the action being like that of nutting one side of a dry board, developing a tendency to warp. The other fact is that these surfaces thus camphor treated, super-saturated it may be with the solvent, seem to lose their elasticity and break easily. Further observation is needed on this last point.

The hardness of the plaster investment of Celluloid is complained of by some as an evil, and there is some difficulty in removing it from frail and delicate pieces. This hardness is in marked contrast with the softness of the plaster investment of rubber cores, when the investing material seems after a few hours to become entirely changed in structure and thoroughly disintegrated. This was commonly believed to be due to the sulphur present in the rubber, but some experiments undertaken a short time ago by the writer at the suggestion of Prof. P. H. Austin, led to the belief that it was the greta pressure of the steam that was the cause of this change in the plaster. Not having had an opportunity of inspecting the plaster used with the steam apparatus we do not know if it is subject to this disintegration or not.

The removal of the plaster from the moulded Celluloid and attached teeth plate, it is said is greatly facilitated by adding a pinch of salt to the water in mixing the plaster, but if this is done the mould should be used at once, as the salt seems to render the plaster soft, after standing awhile. A coat of linseed oil painted over the surface of the mould very greatly facilitates the separation of the plaster. Some difficulty is often found in removing the adherent plaster on the palatal surface of the plate; this the oil almost entirely obviates, making the parting smooth and clear.

The fit of Celluloid to the teeth of the piece seems remarkably close, and it contrasts favorably with rubber in this respect. All are aware that if rubber is cut away for a short distance down upon the neck of a tooth that the line of junction which at the surface seemed clear, is under the surface not closely adapted to the tooth. This seems not to be the case with Celluloid, considerable coming away not revealing any shrinkage within.

